# Porous Silicone Rubber Composite Supported 1,4-Diphenylethynyl Benzene for Hydrogen Absorption with Pd/C Catalyst

**DOI:** 10.3390/ma17081921

**Published:** 2024-04-22

**Authors:** Yu Wang, Tao Xing, Lifeng Yan

**Affiliations:** 1 Department of Chemical Physics, University of Science and Technology of China, Hefei 230026, China; wy1998@mail.ustc.edu.cn; 2 Institute of System and Engineering, China Academy of Engineering Physics, 64 Mianshan Road, Mianyang 621900, China

**Keywords:** hydrogen getter, silicone rubber, porous materials, hydrosilylation

## Abstract

Hydrogen is a dangerous gas as it reacts very easily with oxygen and may explode; therefore, the accumulation of hydrogen in confined spaces is a safety hazard. Composites consisting of unsaturated polymers and catalysts are a common getter, where the commonly used polymer is 1,4- diphenylethynyl benzene (DEB). Silicone rubber (SR) is a good carrier for hydrogen-absorbing materials due to its excellent chemical stability and gas permeability. In this work, polysiloxane, water, and a emulsifier are ultrasonically injected into a uniform emulsion, and the hydrogen getter DEB-Pd/C (Palladium on carbon) is then added. Under the catalysis of platinum (Pt), the cross-linking agent undergoes a hydrosilylation reaction to cross-link polysiloxane in emulsion to form silicone rubber. Then, the water was removed by freeze-drying, and the loss of water constructed a porous frame structure for silicone rubber, thus obtaining porous silicone rubber. The difference in hydrogen absorption performance between porous silicone rubber and ordinary silicone rubber was compared. It was found that, with the increase in water in the emulsion, the porous frame of silicone rubber was gradually improved, and the hydrogen absorption performance was improved by 243.4% at the highest, almost reaching the theoretical saturated hydrogen absorption capacity. Porous silicone rubber was prepared by emulsion mixing, which provided a new idea for further improving the hydrogen absorption performance of silicone rubber.

## 1. Introduction

Hydrogen (H_2_) has strong permeability to materials due to its small volume. It can be captured by the defects in the material, thus generating huge hydrogen pressure inside and causing cracks or even fractures in the material, which is hydrogen corrosion, as shown in Figure S1 [1,2]. Once the hydrogen concentration in the closed environment exceeds 4%, there is even the danger of an explosion. In order to prevent the accumulation of hydrogen in a closed environment, it is necessary to add hydrogen-absorbing materials and use the addition reaction of unsaturated bonds with hydrogen to eliminate hydrogen [3,4].

**Figure 1:**
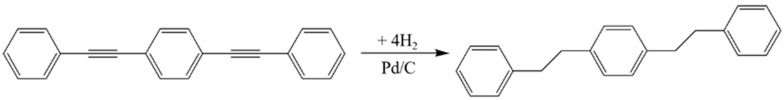
Catalytic hydrogenation of DEB.

Two unsaturated triple bonds in 1,4-diphenylethynyl benzene (DEB) can react with hydrogen under the catalysis of Pd/C, as shown in Figure 1 [5]. DEB-Pd/C is a widely used hydrogen absorber because of its fast reaction rate and high hydrogen absorption capacity [6,7]. However, DEB-Pd/C is a powdery particle that is difficult to work in a closed environment, so its applications are limited. Therefore, a polymer carrier loaded with hydrogen absorbers is needed to solve the problem of its environmental adaptability [8].

Silicone rubber (SR) is a kind of polymer elastomer that is a good carrier of hydrogen-absorbing materials due to its excellent chemical stability and gas permeability [9,10,11]. Generally, SR is obtained by cross-linking liquid polysiloxane, and the most common cross-linking method is use of the hydrosilylation reaction. The hydrosilylation reaction is an addition reaction between the vinyl group on polysiloxane and the functional Si-H groups on the oligomer under the catalysis of the organometallic complexes of Pt or Rh [12]. The main chain of the obtained silicone rubber is composed of silicon and oxygen atoms alternately, and the organic functional groups in the side chain are attached to both sides of the silicon atoms after modification [13,14,15,16]. This structure makes it semi-organic or semi-inorganic [17,18]. However, when silicone rubber is used as the carrier of a hydrogen absorber, it is necessary to solve the problem of hydrogen being difficult to diffuse inside the rubber, as, otherwise, the hydrogen will only react with the hydrogen absorber in a certain range on the outer surface of silicone rubber, resulting in lower hydrogen absorption efficiency, while the actual hydrogen absorption amount is far lower than the theoretical hydrogen absorption amount [19,20]. Therefore, it is necessary to build a porous frame structure for silicone rubber to accelerate the diffusion of hydrogen inside the rubber [20].

In this work, the hydrogen absorber DEB-Pd/C has been synthesized by crosslinking vinyl PDMS using poly(methylhydrosiloxane) as the crosslinker in aqueous emulsion. A series of silicone rubbers with different water contents in the emulsion were prepared, and the differences in hydrogen absorption properties between porous silicone rubber and ordinary silicone rubber were compared, as well as the differences in hydrogen absorption properties of silicone rubbers with different water contents in the emulsion. It was found that porous silicone rubber greatly improved the hydrogen absorption properties of the system, and, with the increase in water content, the hydrogen absorption properties of porous silicone rubber also increased and almost reached the theoretical saturated hydrogen absorption capacity. Porous silicone rubber was prepared by emulsion mixing, which provided a new idea for further improving the hydrogen absorption performance of silicone rubber. The porous structure and their homogenous distribution will help the hydrogen diffusion and the reaction with the reactive groups inside the material.

**Figure 2:**
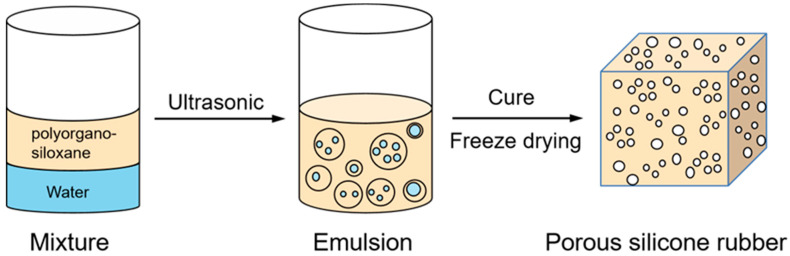
Formation of Porous Silicone Rubber.

Figure 2 shows the synthesis route for the porous silicone rubber.

## 2. Materials and Methods

### 2.1. Materials

DEB was purchased from ANV CHEM CO., Ltd. (Tianjin, China) and Pd/C was purchased from Aladdin Chemistry Co., Ltd. (Shanghai, China), while other chemicals were all purchased from Maclin Chemical Inc. (Shanghai, China). The Pd/C catalyst needs to be dried in a 70 °C vacuum drying oven for 12 h to remove excess water inside. All other chemicals were used without further purification. Deionized water (18.2 MΩ·cm) was generated via a Milli-Q system (Millipore, St. Louis, MO, USA). Vinyl PDMS (M_W_ 28000), poly(methylhydrosiloxane) (15–40 mPa·s (20 °C)).

### 2.2. Preparation of DEB-Pd/C Getter

Vinyl PDMS, water, and an emulsifier were ultrasonicated in an ultrasonic cell crusher to obtain a uniform white emulsion, and hydrogen absorber DEB-Pd/C and cross-linking agent poly(methylhydrosiloxane) were then added subsequently [21]. Under the catalysis of platinum (Pt), the cross-linking agent took place in a hydrosilylation reaction to cross-link the continuously polymerized siloxanes in the emulsion to form silicone rubber, as shown in Figure S2. Then, the water in the emulsion was removed by freeze-drying, and a porous frame was constructed for the silicone rubber. A series of silicone rubbers with different water contents in the emulsion were prepared, and the differences in hydrogen absorption properties between porous silicone rubber and ordinary silicone rubber were compared, as well as the differences in the hydrogen absorption properties of silicone rubbers with different water contents in the emulsion. In brief, 0.75 g of DEB and 0.25 g of Pd/C were put into a mortar and fully ground to obtain the getter of DEB-Pd/C. Part of DEB-Pd/C was treated by catalytic hydrogenation, and H-DEB-Pd/C was obtained for subsequent testing.

### 2.3. Preparation of Polysiloxane Emulsion

The amounts of 1 g of vinyl PDMS, 0.8 g of deionized water, and 0.2 g of triton X-100 (its chemical structure is shown in Figure S3) were added to the reactor. Two drops of poly(methylhydrosiloxane) were added as a cross-linking agent, and they were then placed into an ultrasonic cell pulverizer for ultrasonic treatment until a uniform emulsion was formed. At this time, the mass ratio of polysiloxane, water, and emulsifier in the emulsion was 5:4:1, and the sample was recorded as W4-SR. Then, two kinds of emulsions with different water contents were prepared, and the deionized water in the above step was changed from 0.8 g to 1.2 g and 1.6 g. At this time, the ratio of polysiloxane, water, and emulsifier was 5:6:1 and 5:8:1, respectively, and the samples were recorded as W6-SR and W8-SR, respectively. In addition, without adding water, only 1 g of vinyl PDMS, 0.2 g of triton X-100, and two drops of poly(methylhydrosiloxane) were evenly mixed as the control group, and the sample was recorded as W0-SR.

### 2.4. Preparation of Porous Silicone Rubber

An amount of 0.25 g of the prepared DEB-Pd/C getter was used, and two drops of 1,3-divinyl-1,1,3,3-tetramethyldisiloxane platinum (0) as a crosslinking catalyst were added to all the above four samples under stirring with a magnetic stirrer at 1000 r/min for 4 h until the emulsion was uniform. The solution was allowed to cross-link at 65 °C for 72 h, and it was then cooled down to room temperature. The above steps were repeated for all four samples to obtain porous silicone rubber with different pore sizes.

### 2.5. Gel Fraction

In general, the gel fraction of a polymer network that has been crosslinked is measured through dissolving in toluene and measuring the gel contents. After immersing the molded film in toluene for 24 h, the undissolved material was filtered off, dried in an oven at 80 °C, and then the gel content was weighed. The gel content expressed as the gel fraction (in %) was calculated according to the relationship (W_1_/W_0_) × 100, where W_0_ is the weight of the crosslinked polymer network and W_1_ is the weight of the same network after toluene extraction and drying.

### 2.6. Hydrogen Absorption Test

**Figure 3:**
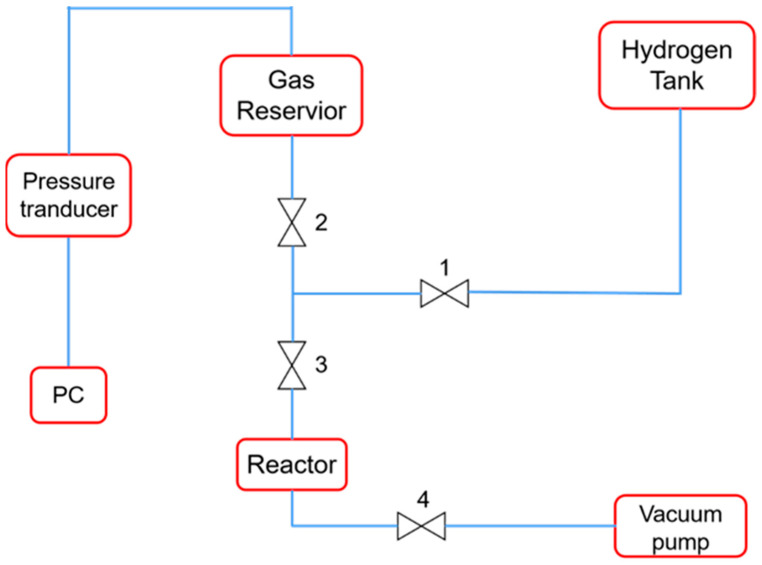
Schematic diagram of hydrogen absorption capacity testing device.

A homemade apparatus is shown in Figure 3 for conducting absorption tests in pure H_2_ atmospheres. By using an L300500i helium mass spectrometer leak detector (Leybold, Koln, Germany), it was determined that the leakage rate of the apparatus was less than 10^−9^ Pa m^3^/s, which was suitable for the test. The sample was placed in a stainless-steel reactor (Xuwei Technology Inc., Chengdu, China) with a 100 mL volume; the reactor was then closed and a vacuum was applied to the apparatus (Valve 1 off, with 2, 3, and 4 on). When the internal pressure of the gas reservoir was pumped to 100 Pa, the vacuum pump and the reactor were disconnected (valves 4 and 3 off), and H_2_ from the hydrogen tank was slowly introduced to the device (Valves 1 and 2 on) until the internal pressure reached 200 KPa. Then, the reactor was reconnected (Valve 1 off and 3 on) and H_2_ was introduced into the reactor. The data acquisition software (SUPY1.0, 2015, Simingte Inc., Hangzhou, China) was then used to record and analyze the data. The pressure change was recorded as a function of time, and the capacity (volume of hydrogen absorbed per SR or catalyst weight, mL/g) was calculated from the pressure drop according to the ideal gas equation (Equation (1)):

(1) $$Capacity=\frac{(P_{1}V_{1}-P_{2}\left(V_{1}+V_{2}-V_{3}\right))\times 22400}{mRT}$$

where *m* is the weight of the SR or the catalyst, *T* is temperature, *R* is the ideal gas constant, and *V*_1_ is the volume of the gas reservoir and tubing between Valve 1 and two containers (gas reservoir and reactor), which was calibrated to be 312.0 mL. *V*_2_ is the volume of the reactor, which was calibrated to be 114.6 mL, and *V*_3_ is the volume of the sample, which was about 0.8 mL when a 1.0 g sample was tested.

### 2.7. Characterization

Fourier transform infrared spectroscopy (FTIR, Nicolet 6700, transmission, Thermo Scientific, Waltham, MA, USA) was used to describe the difference of functional groups between polysiloxanes; additionally, the wavelength range was 400–4000 cm^−1^ and the absorption of the samples was tested.

For the ^1^H NMR (BRUKER 400M, Billerica, MA, USA), a small amount of DEB before and after hydrogenation was dissolved in deuterated dimethyl sulfoxide (DMSO). Because Pd/C was mixed in the hydrogenated DEB, an organic filter membrane was needed to filter out the insoluble Pd/C, which was used to characterize whether DEB could be completely hydrogenated.

For the scanning electron microscope (SEM, XL30 FEG-SEM Philips, FEI, Hillsboro, OR, USA), the sample was attached to the sample table on a conductive tape and vacuum coated with a coating instrument to observe the micro-morphology of porous silicone rubber.

X-ray photoelectron spectroscopy (XPS, Kratos Axis supra+, Kratos Analytical, Manchester, UK), using an Al kα monochromatic X-ray source and operating voltage of 15 kV, characterized the elemental composition and state of the sample.

For the thermogravimetric analysis (TG, SDT Q600, TA Instruments, New Castle, DE, USA), the thermogravimetric curve of the sample was tested in N2 atmosphere, and the heating rate was 20 °C/min. The thermogravimetric curve of the sample was obtained in a range from 80 °C to 800 °C to characterize the thermal stability of the sample.

## 3. Results and Discussion

### 3.1. Characterization of DEB before and after Hydrogenation

**Figure 4:**
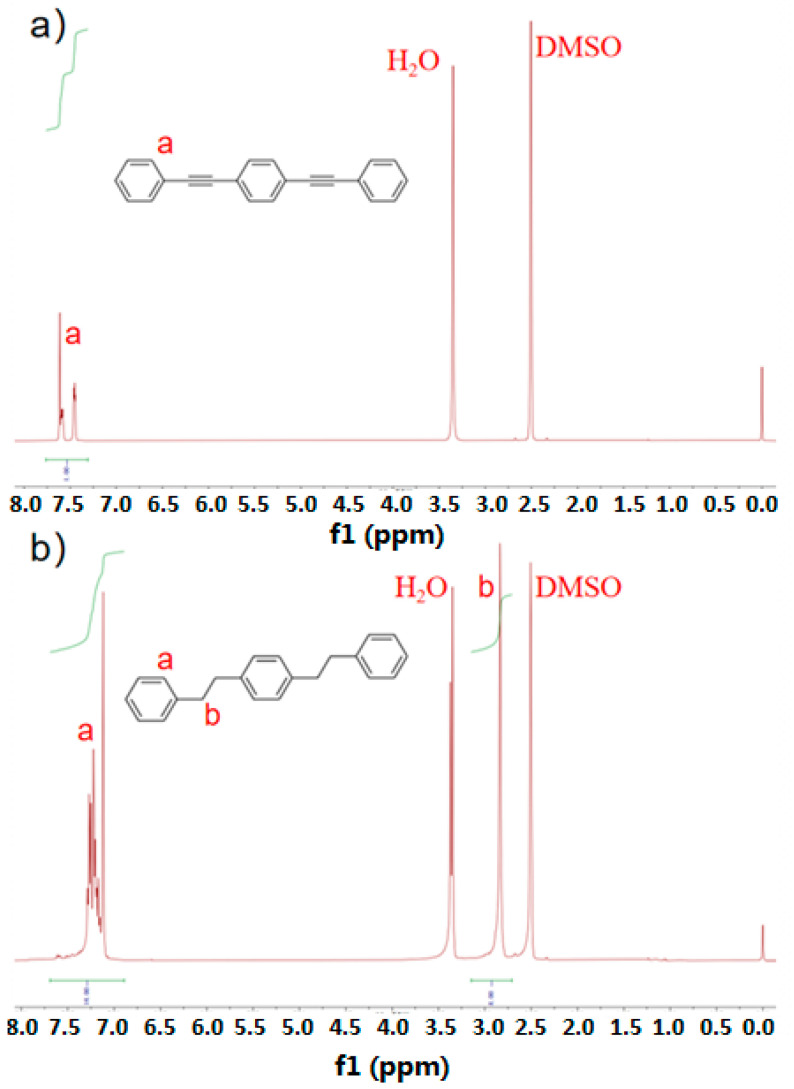
^1^H NMR spectra of (**a**) 1,4-diphenylethynyl benzene (DEB) and (**b**) hydrogened 1,4-diphenylethynyl benzene (H-DEB).

In order to characterize the changes in the molecular structure of DEB before and after the hydrogen absorption reaction, and to distinquish whether it is completely hydrogenated, DEB was analyzed by ^1^H NMR. As shown in Figure 4, two samples were dissolved by deuterated DMSO, and a new peak appeared at 2.8 ppm after DEB was hydrogenated to H-DEB, indicating that the C≡C bond in DEB was not partially hydrogenated and that the C≡C bond was completely hydrogenated to CH_2_-CH_2_ [5].

**Figure 5:**
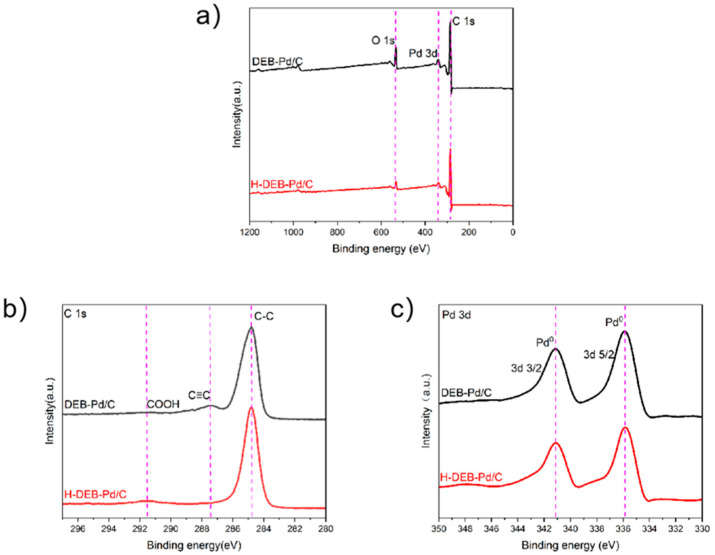
(**a**) XPS full scanning spectrograms of Deb-PD/C and H-DEB-Pd/C; (**b**) C 1s spectrogram; (**c**) Pd 3d spectrogram.

In order to further explore the changes in the elemental composition and chemical bonding state of getter DEB-Pd/C before and after catalytic hydrogenation, an XPS analysis was carried out on DEB-Pd/C. As shown in Figure 5a, the XPS full-scanning spectrum of DEB-Pd/C before and after hydrogenation shows that there are no new or reduced peaks in the sample before and after hydrogenation, but the peak intensity of O 1s is obviously reduced, which is due to the fact that the oxygen-containing groups on activated carbon are also reduced by hydrogen during DEB hydrogenation. As shown in Figure 5b, C 1 s scanning curves of DEB-Pd/C and H-DEB-Pd/C are shown. It can be seen that, in H-DEB-Pd/C, the peak intensity of C≡C is obviously reduced or even disappears, which indicates that the C≡C bond in DEB can be completely hydrogenated into a C-C bond instead of being partially hydrogenated. As shown in Figure 5c, the Pd 3d scanning curves of DEB-Pd/C and H-DEB-Pd/C are shown. It can be seen that there is no new peak and no disappearance of the old peak, while the peak intensity has not changed significantly, indicating that the Pd catalyst only plays a catalytic role before and after catalytic hydrogenation and will not participate in the reaction [22].

### 3.2. Characterization of Porous Silicone Rubber

**Figure 6:**
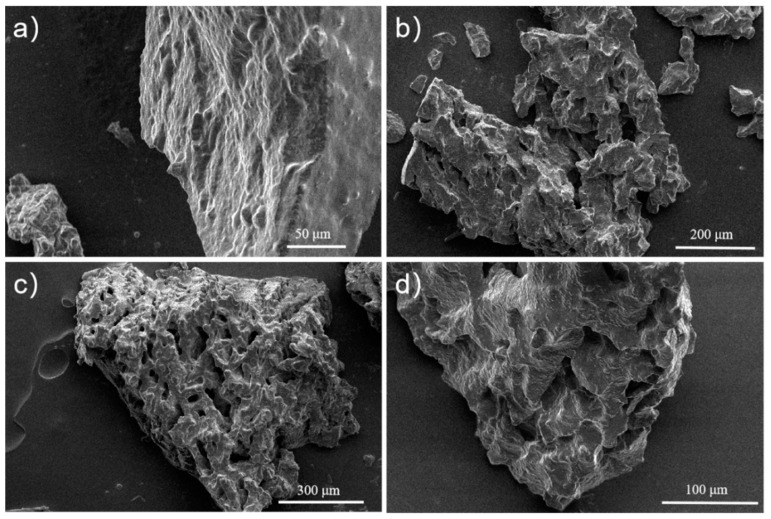
SEM images of cross sections of (**a**) W0-SR; (**b**) W4-SR; (**c**) W6-SR and (**d**) W8-SR.

The porous microstructure of silicone rubber composites prepared by adding different moisture emulsions was observed by SEM. As shown in Figure 6a, the cross-section of W0-SR is flat and there are no small holes; Figure 6b is the SEM image of the W4-SR cross-section. It can be seen that pores begin to appear on the surface of SR at this time, but the distribution density of the pores is not large and the pore size is also small. Figure 6c is the SEM image of the cross-section of W6-SR. Compared with W4-SR, it is found that the density of pores is greatly increased at this time, the size of most pores is maintained between 30 and 40 μm, and the porous framework of SR begins to appear. Figure 6d is the SEM image of the cross-section of W8-SR. Compared with W6-SR, the pore density has not increased significantly at this time, but the pore size has obviously increased, and most of the pore sizes are between 40 and 50 μm. It is also shown that W4-SR does not have a good porous structure, while W6-SR and W8-SR have a complete porous framework with a uniform distribution of pores. The gel fractions of the sample are 98%, 81%, 83%, and 84% for W0-SR, W4-SR, W6-SR, and W8-SR, respectively. 

**Figure 7:**
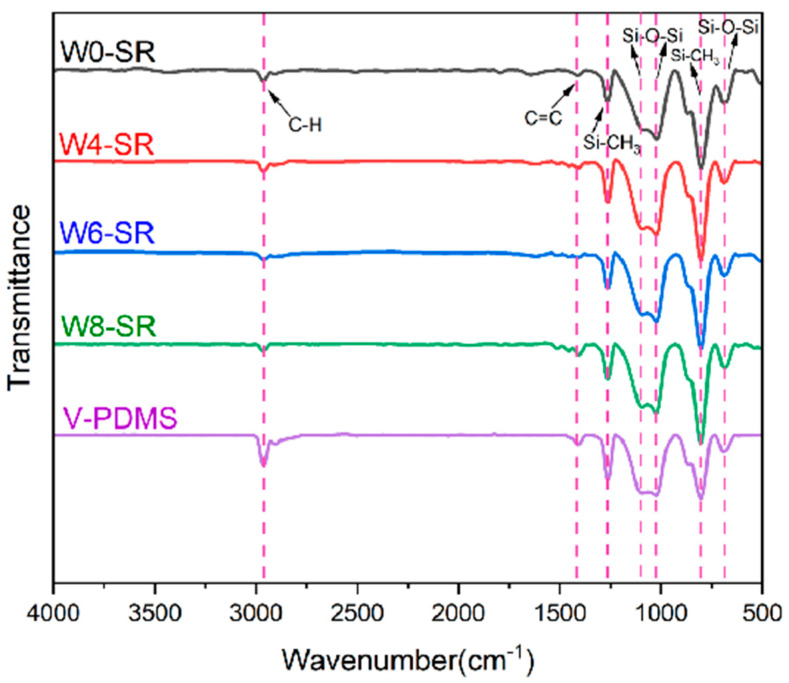
Infrared spectra of V-PDMS and the silicone rubber composites.

FT-IR was used to explore the silicone rubber composites made from emulsions containing different moisture levels and the influence of moisture on its cross-linking structure. As shown in Figure 7, the infrared spectra of vinyl PDMS and four kinds of silicone rubber composites without getter DEB-Pd/C are shown. It is found that the absorption peaks are basically the same, and there is no obvious appearance of new peaks or a disappearance of old ones. There are obvious absorption peaks at 1261 cm^−1^ (tensile vibration of Si-C), 1101 cm^−1^ and 1018 cm^−1^ (tensile vibration of Si-O-Si), 798 cm^−1^ (bending vibration of Si-C), and 690 cm^−1^ (bending vibration of Si-O-Si) [16]. However, with the C=C stretching vibration peak at 1410 cm^−1^, it can be found that the peak intensity of these five samples roughly follows the law of weak to strong from top to bottom [23,24]. The C=C bond is the site where V-PDMS is cross-linked, indicating that adding water to the emulsion will affect the cross-linking effect of polysiloxane. The size of the as-formed emulsion has a direct relation to the water amount and results in the variation in the crosslinking reaction. Therefore, the more water that is added, the worse the cross-linking effect of the polysiloxane is.

**Figure 8:**
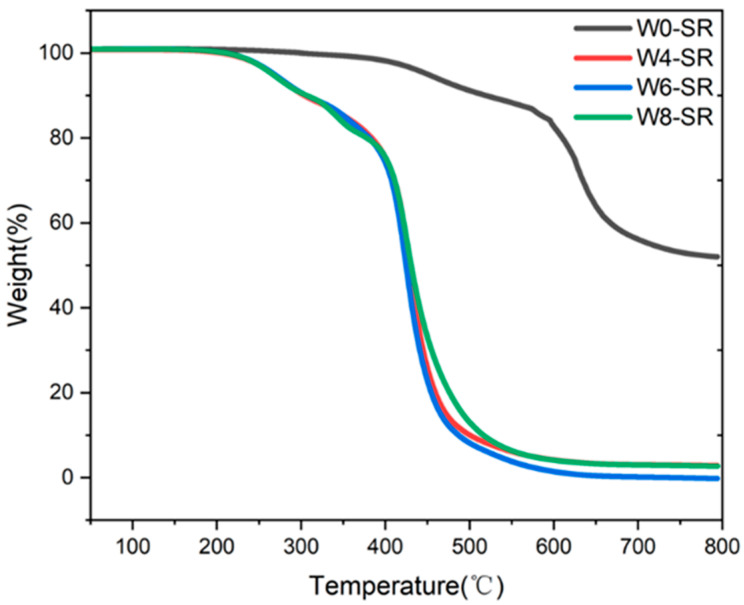
TG curves of W0-SR, W4-SR, W6-SR and W8-SR.

Thermogravimetric analysis (TGA) was used to analyze the differences in the thermal stability of the silicone rubber composites prepared from emulsions containing different levels of moisture. As shown in Figure 8, the decomposition temperature of W0-SR is in the range of 300–700 °C, while the decomposition temperatures of the other three kinds of W4-SR, W6-SR, and W8-SR with water in the emulsion are in the range of 200–500 °C. Additionally, their thermal stability is basically the same, indicating that adding water in the emulsion will lead to a decrease in the thermal stability of the prepared silicone rubber, while adding water in the emulsion. The results also reveal that, after crosslinking without adding water, the crosslinked degree is higher than the other samples. 

**Figure 9:**
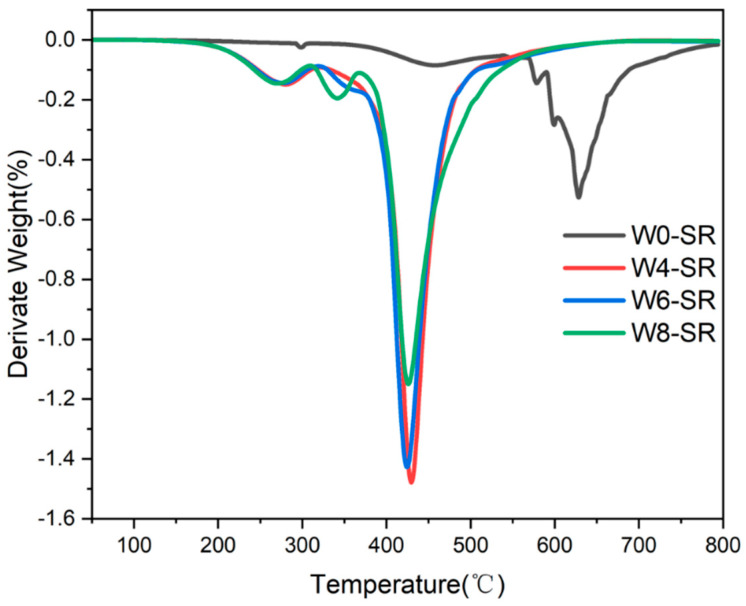
DTG curves of W0-SR, W4-SR, W6-SR, and W8-SR.

Figure 9 shows the DTG curve corresponding to the TGA curve, and it can be seen that the thermal weight loss of four kinds of silicone rubber composites has three stages. The first stage (200–315 °C) is the fracture of the Si-C cross-linking bond formed by hydrosilylation, which leads to a decrease in the molecular weight of silicone rubber. In the second stage (315–375 °C), the crosslinking of silicone rubber was destroyed, and the decomposed poly(methylhydrosiloxane) was decomposed, resulting in the relative thermal weight loss. The third stage (375–550 °C) is the most important thermogravimetric stage, and it is caused by the random breakage of Si-O-Si bond in poly(methylhydrosiloxane), as the polymer gradually becomes small molecules and volatilizes [23,25]. Compared with the other three sample of W4-SR, W6-SR, and W8-SR, the third stages of W0-SR occur at higher temperatures (550–720 °C). Clearly, the crosslinking of silicone rubber makes it difficult to destroy, and the decomposition of polysiloxane is difficult to cause, also indicating that the crosslinking effect of silicone rubber without water in emulsion is better and that the addition of water will affect the crosslinking of polysiloxane.

### 3.3. Hydrogen Absorption Properties of Porous Silicone Rubber

**Figure 10:**
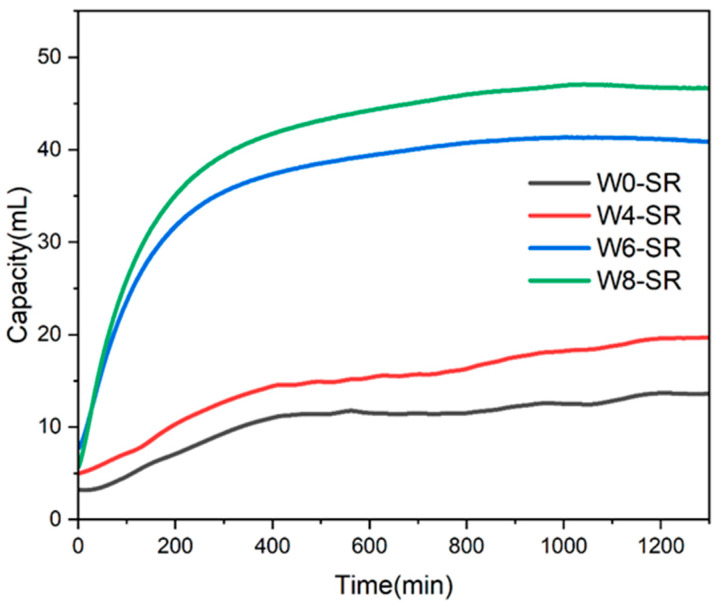
Hydrogen absorption curves of W0-SR, W4-SR, W6-SR, and W8-SR.

In order to explore the improvement of the hydrogen absorption performance of porous silicone rubber composites, a series of silicone rubber composites made of emulsions containing different moisture levels was prepared, and their hydrogen absorption was tested. Each sample contains 0.1875 g of DEB, and each DEB molecule contains two C≡C bonds, which can absorb four hydrogen molecules; therefore, the theoretical saturated hydrogen absorption is calculated to be 52.2 mL (hydrogen under standard conditions). As shown in Figure 10, the hydrogen absorption rates of W0-SR and W4-SR are both slow at the beginning, especially after 500 min. Due to the lack of a porous structure, it is difficult for W0-SR to diffuse into the silicone rubber, and the hydrogen absorption is basically saturated, while W4-SR is still slowly absorbing hydrogen after 500 min, which is due to the certain porous structure promoting the diffusion of hydrogen in the silicone rubber, as shown in Table 1. Compared with W0-SR, the hydrogen absorption capacity at 1300 min is increased by 44.9%; additionally, compared with the theoretical hydrogen absorption capacity, the achievement rates of the hydrogen absorption capacity are 26.1% and 37.3%, respectively. Compared with W0-SR and W4-SR, W6-SR has a greatly improved hydrogen absorption rate and capacity, and the initial hydrogen absorption rate is also faster, as adding more water into the emulsion can form an interpenetrating network structure between the pores in the molded silicone rubber. Meanwhile, it is still difficult to diffuse hydrogen in W4-SR interior interior as the pores are far apart, and so the hydrogen absorption capacity at 1300 min is 40.8 mL. Compared with W6-SR, the hydrogen absorption rate of W8-SR is faster, and the hydrogen absorption capacity is also increased by 14.5%. The achievement rate of the saturated hydrogen absorption is 89.5%, and the improvement range is limited. Compared with W0-SR, the hydrogen absorption capacity at 1300 min is 46.7 mL, which is increased by 243.4%, further improving the hydrogen absorption capacity and hydrogen absorption rate. The as-prepared porous silicone rubber composite DEB-Pd/C materials showed much higher performances that the previously reported SGO-supported palladium (Pd) hydrogen getters [26].

## 4. Conclusions

In this work, under the catalysis of platinum, the cross-linking agent poly(methylhydrosiloxane) created a hydrosilylation reaction to cross-link V-PDMS to form silicone rubber, and the water was then removed by freeze-drying to form a porous silicone rubber hydrogen-absorbing material. The porous structure of the silicone rubber was adjusted by adjusting the moisture in the emulsion, and a series of silicone rubber hydrogen absorbing materials with different porous structures were manufactured by this method. It was found that the porous structure of the as-prepared silicone rubber was obvious, and the porous framework became more and more complete with the increase in water in the emulsion. The thermogravimetric curves show that the thermal stability of porous silicone rubber is obviously lower than that of W0-Sr. The hydrogen absorption properties of porous silicone rubber and W0-SR were compared by a hydrogen absorption test device. It was found that the hydrogen absorption rate and capacity of W6-SR and W8-SR were greatly improved from W6-SR, the maximum hydrogen absorption capacity was increased by 243.4% at 1300 min, and the actual hydrogen absorption rate was also increased from 26.1% of W0-SR to 89.5% of W8-SR, providing new ideas for the preparation of porous hydrogen-absorbing material.

## Figures and Tables

**Table 1:** Hydrogen absorption test results of W0-SR, W4-SR, W6-SR, and W8-SR.

Sample	Hydrogen Absorption	Improved Capacity	Theoretical Hydrogen Absorption	Yield Rate
W0-SR	13.6 mL	0%	52.2 mL	26.1%
W4-SR	19.7 mL	44.9%		37.3%
W6-SR	40.8 mL	200.0%		78.2%
W8-SR	46.7 mL	243.4%		89.5%

## Data Availability

Data is contained within the article.

## Supplementary Materials

The following supporting information can be downloaded at: https://www.mdpi.com/article/10.3390/ma17081921/s1, Figure S1. Mechanism of hydrogen corrosion of materials, Figure S2. The chemical structure of triton X-100, Figure S3. Mechanism of hydrosilylation.
