# Supplementary material for: Porous Silicone Rubber Composite Supported 1,4-Diphenylethynyl Benzene for Hydrogen Absorption with Pd/C Catalyst
Source: Materials (Basel). 2024 Apr 22;17(8):1921. doi: 10.3390/ma17081921 (PMC11051936; doi:10.3390/ma17081921)
Supplement: Supplementary file 1 [file materials-17-01921-s001.zip › materials-2950937-supplementary.pdf]

Supporting Information:

Porous Silicone Rubber Composite Supported 1,4-Diphenylethynyl Benzene for Hydrogen Absorption with Pd/C Catalyst

Yu Wang [1], Tao Xing [2,*], and Lifeng Yan [1,*]

[1] Department of Chemical Physics, University of Science and Technology of China, Hefei 230026, China; wy1998@mail.ustc.edu.cn (Y.Wang); and lfyan@ustc.edu.cn (L. Yan)

[2] Institute of System and Engineering, China Academy of Engineering Physics, 64 Mianshan Road, Mianyang 621900, China; 412xingt@caep.cn

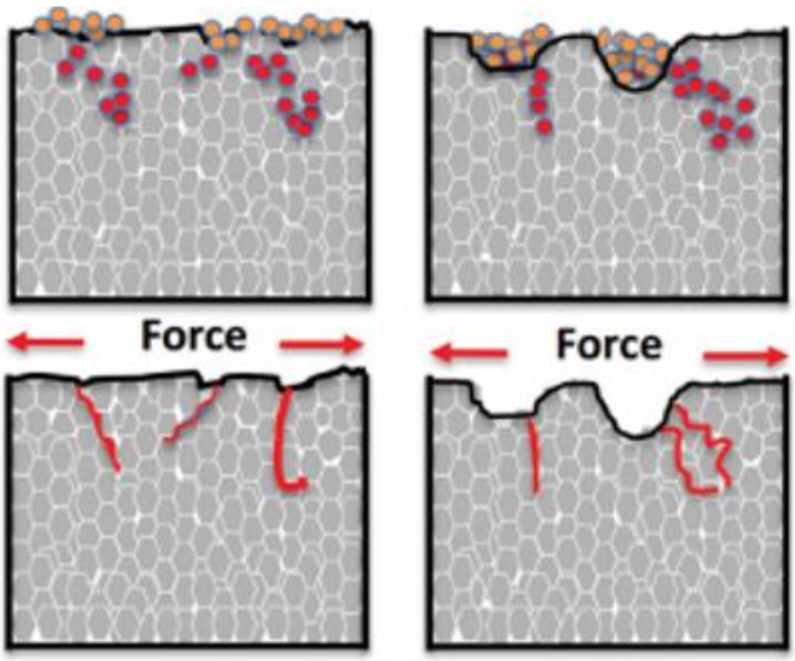

Figure S1. Mechanism of hydrogen corrosion of materials.

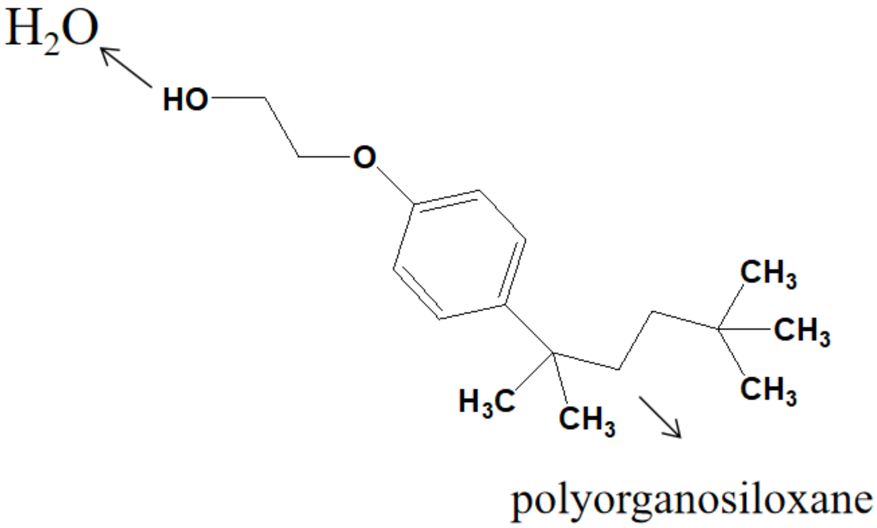

Figure S2. Structural formula of triton X-100.

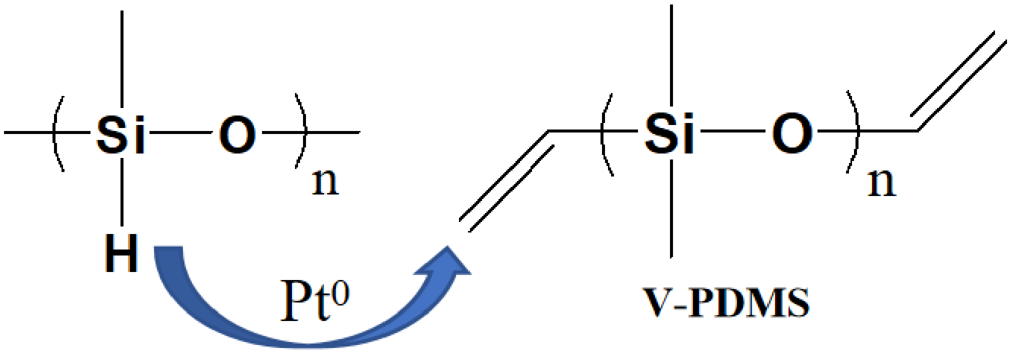

Figure S3. Mechanism of hydrosilylation.
